# Assessing the need for a protocol in monitoring weight loss and nutritional status in orthognathic surgery based on patients experiences

**DOI:** 10.4317/jced.53354

**Published:** 2017-02-01

**Authors:** Muhammad Ruslin, Hannah Dekker, Dirk B. Tuinzing, Tymour Forouzanfar

**Affiliations:** 1 Department of Oral and Maxillofacial Surgery Faculty of Dentistry Hasanuddin University, Makassar, Indonesia; 2 Department of Oral and Maxillofacial Surgery/Oral Pathology, VU University Medical Center/Academic Center for Dentistry Amsterdam (ACTA), Amsterdam, The Netherlands

## Abstract

**Background:**

To investigate retrospectively the orthognathic surgery (OGS) patients experience in weight loss and the influence of gender, age, duration of the surgical procedure, length of hospital stay, location of surgery and use of intermaxillary fixation (IMF) or without IMF on postoperative weight loss.

**Material and Methods:**

A total of 4487 patients treated by OGS where all patients visited the outpatient clinic one, three and six weeks after the surgical procedure. After six weeks, patients filled out a questionnaire in which weight loss was addressed. The patients were asked to give an estimate of their experiences weight loss. The population was first divided in two groups weight loss and no weight loss.

**Results:**

In the weight loss group there is no significant difference in weight loss between patients with IMF and patients without IMF. In the weight loss group there were significantly more females then males. Further, in the subgroup IMF the operation time was significantly longer compared with the subgroup without IMF. The other parameters including age and hospital stay were not different in the groups.

**Conclusions:**

IMF in orthognathic treatment does not result in a difference self-reported loss of body weight compared to patients without IMF. Treatment protocols should include pre- and post-operative dietician consultations and possible indications for medical nutrition and vitamins.

** Key words:**Assessing, protocol, weight loss, experiences, orthognathic surgery.

## Introduction

Double-jaw osteotomies play an important role in the maxillofacial surgery (1). Recuperation following osteotomies is a complex process for the patient and requires the resolution of postoperative sequel, such as, nausea, swelling, pain, discomfort, uncomfortable oral function, and decreased activity levels (2,3). Surgery on its own is an additional trauma to the patient that is why nutrition may play a more important role in surgical patients than in healthy subjects. Good and balanced nutrition is an important cofactor for decreased mortality and morbidity in surgical cases (4). Proper wound healing requires specific nutritional requirements which must be met to allow optimal repair of the tissues, it is important for the surgeon to have and understanding of the pre-surgical nutritional state of the patients in order to adequately plan for and prevent any complications which might arise due to a poor healing of the tissues (5). Guo *et al.* reported poor nutrition plays an important part in the development of postoperative complications, and perioperative nutritional support of patients with oral and maxillofacial cancer must be properly managed (6). The patients sustaining maxillofacial trauma, disease or deformity presents with unique nutritional problems, especially during the postoperative period, by providing a nutritionally adequate diet in the preoperative and immediate postoperative period and during convalescence, complications can be reduced and healing improved (7).

In several osteotomy cases intermaxillary fixation (IMF) is applied. IMF is one of the modalities used before the utilization of mini-plates to stabilize and promote the healing of fractured facial bones in cases of trauma or orthognathic surgery (OGS) (8). Weight loss has been noted as one of the major side effects of IMF in patients with mandibular fractures (8) or OGS (9). Furthermore, previous studies have suggested IMF for diet control (9-12). Patients undergoing OGS and subsequent IMF are at risk for malnutrition and substantial weight loss. Eventually this may lead to impaired bone and wound healing and thus to a deteriorated overall functional recovery (13).

In the literature there is a lack of systematic documentation of the weight loss experienced by patients during the first few weeks or months or of the time required for postoperative recovery following OGS. The aim of the present study was to investigate retrospectively the OGS patients experience in weight loss and the influence of gender, age, duration of the surgical procedure, length of hospital stay, location of surgery and use of IMF or without IMF on post-operative weight loss.

## Material and Methods

At the Department of Oral and Maxillofacial Surgery/Oral Pathology, VU University Medical Center and Academic Center for Dentistry Amsterdam, The Netherlands, all of patients undergoing OGS had been registered in a database since January 1970. All patients visited the outpatient clinic one, three and six weeks after the surgical procedure. After six weeks, patients filled out a questionnaire in which weight loss was addressed. The patients were asked to give an estimate of their experiences weight loss. Data on actual pre- and post-operative body weight were not registered. At the time of the present study the database consisted of 4487 patients.

Anthropometric variables included gender, age, duration of the surgical procedure, length of hospital stay, location of surgery (maxilla, mandible and bi-maxillary), use of IMF, and experienced weight loss as registered in the questionnaire. Do patients after OGS experience weight loss and what are the patients that typically experience correlate then with gender, age, duration of the surgical procedure, length of the hospital stay, location of surgery and use of IMF or without IMF? The patients were divided into 2 groups include weight loss and without weight loss after surgery.

The statistical analyses were conducted with the Statistical Package for Social Sciences (SPSS) version 20.0.

## Results

**Figure 1:**
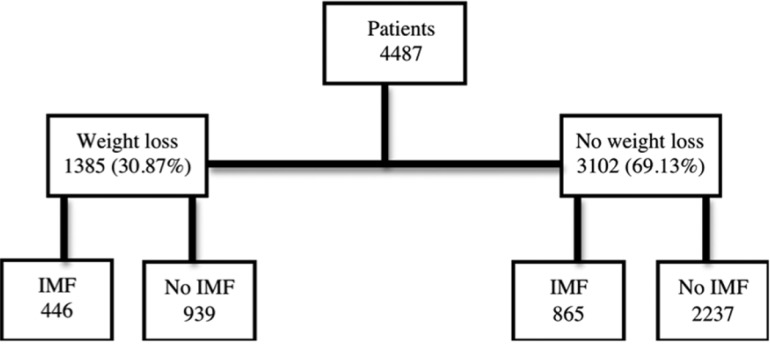
Patient distribution. The study population consisted of 4487 patients. The group with experienced weight loss consisted of 1385 (30.87%) subjects and without weight loss consisted of 3102 (69.13%).

The study population consisted of 4487 patients. As shown in figure 1 and  Table 1 the group with experienced weight loss consisted of 1385 (30.87%) subjects. Those patients, 939 had no IMF and 446 subjects had IMF. There were significantly more females experience weight loss then males (*p* < 0.05). There were no differences in weight loss between the groups. In the IMF group the surgery time was significantly higher (*p* < 0.05), which seems logical as significant more patients underwent a bi-maxillary surgery in this group. The other parameters were not significant.

**Table 1:**
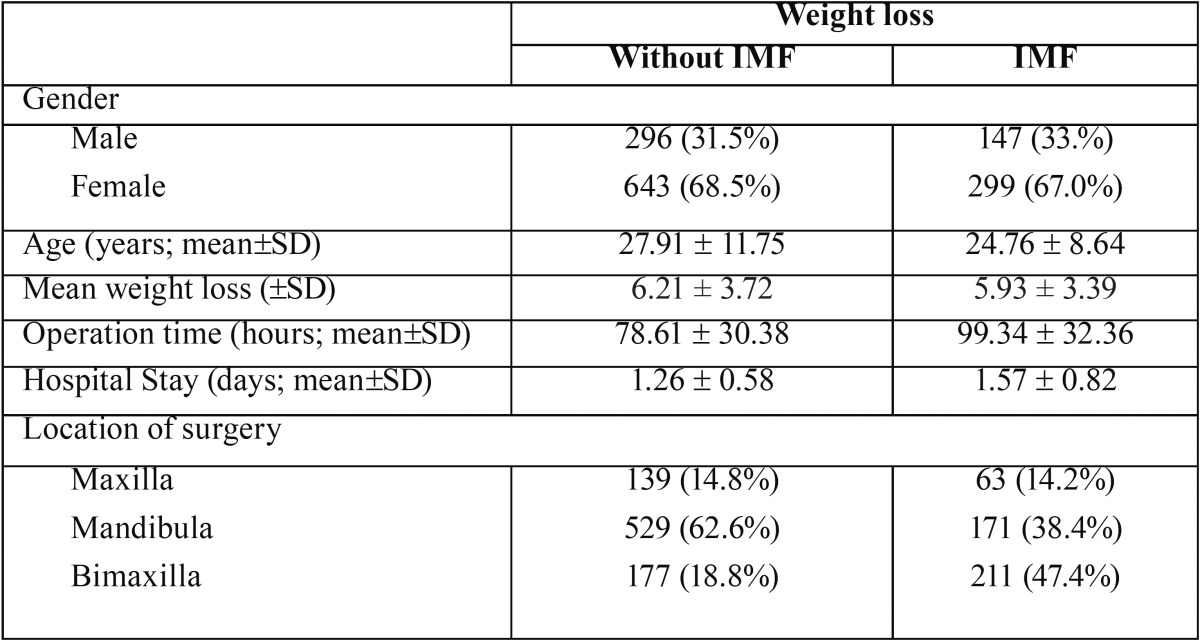
The group with experienced weight loss consisted of 1385 (30.87%) subjects. Those patients, 939 had no IMF and 446 subjects had IMF.

In  Table 2 the patients without weight loss are demonstrated. Those 3102 (69.13%) subjects, 2237 had no IMF whereas there were 865 with IMF. Again the surgery time was significantly longer in IMF group compared to the no IMF group. There were more patient with a bi-maxillary surgery in the IMF group (*p* < 0,05). Other parameters were not significant different.

**Table 2:**
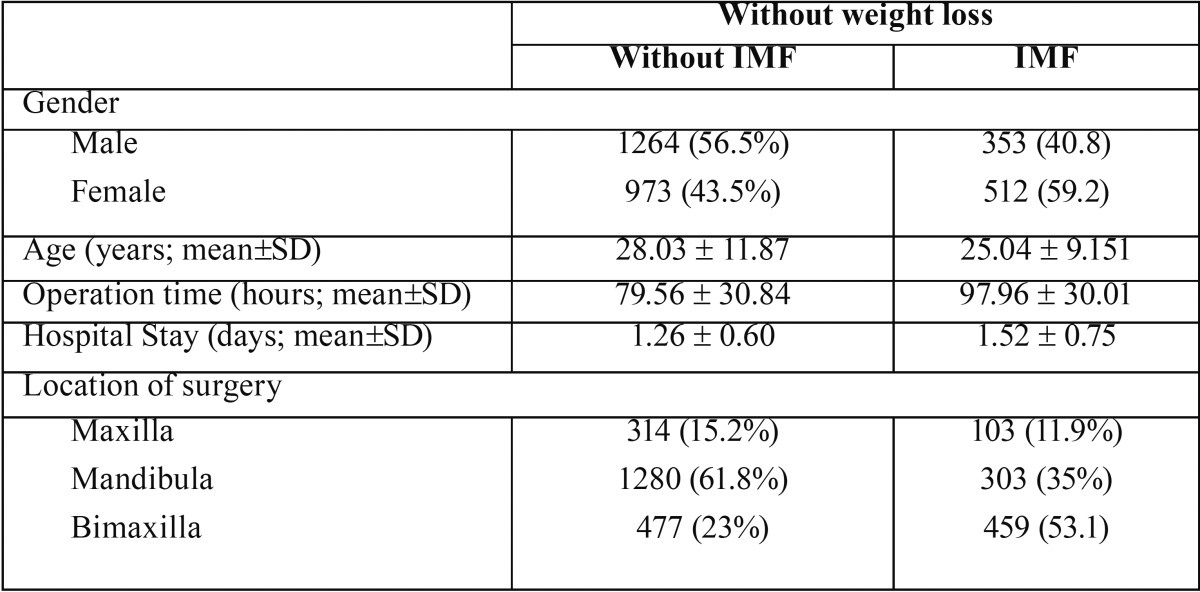
The patients without weight loss are demonstrated. Those 3102 (69.13%) subjects, 2237 had no IMF whereas there were 865 with IMF.

## Discussion

Orthognathic surgery appears to have the largest impact on appearance and chewing, followed by comfort (14). Slightly, higher percentage of subjects report difficulty with food collection in the surgical sites and bad taste/breath after OGS (15). Almost 50% of the OGS patients report that these issues cause at least some concern postoperatively (2). Several authors note that patients undergoing OGS, especially bi-maxillary surgery are at risk of malnutrition and substantial weight loss by the use of IMF (13,16,17). Phillips *et al.* report that problems with eating, chewing, and mouth opening took the longest to resolve, at approximately 6 to 8 weeks, subsequently leading to weight loss (2). As there is still a lack of information on this important issue the present retrospective study was performed. The main interest of this study is to investigate in the influence of weight loss in patients who underwent OGS. Furthermore, a comparison was performed between patients who received IMF and patients who did not received IMF.

There are 30.87% of all patients report weight loss in this study underwent with IMF or without IMF, that IMF does not lead to significant weight loss compared to patients without IMF, which proves to be in line with the study of Worrall (17), and Behbehani *et al.* that report IMF for diet control is not effective for long-term weight reduction and should only be used for a limited period of time to initiate weight reduction (9). Furthermore, in line with the study of Antila *et al.* the impaired oral intake due to IMF does not report interfere significantly with zinc status as estimated by mononuclear cell, polymorphonuclear cell and serum zinc level (18). The reduction in body weight and anthropometric indices in the relatively short fixation period may be clinically significant in some patients. On the other hand Kuvat *et al.* (13) demonstrate the female patients who underwent double jaw osteotomies whose weight decreased significantly (13). In this study, there are no differences in weight loss between females and males however we cannot demonstrate any relation between weight loss and the use of IMF.

The present study has several shortcomings that the weight loss was not measured but noted by the patients in a questionnaire. Many methods are available to estimate body composition, such as body mass index, fat mass and fat-free mass, but few are applicable to the clinical environment in which most oral maxillofacial surgeons work. Determining nutritional status can also be monitored with a comprehensive clinical tool based on a medical and physical assessment (19). Further the data were analyzed retrospectively. Both shortcomings can lead to information bias. The study population consisted of 4487 patients which is the largest population described in the literature concerning this subject. Therefore according to the authors, conclusion can be drawn from the results of the presented study. Patients undergoing OGS experience some degree of weight loss, which can not to be related to age, type and duration of surgery, length of hospital stay, and use of IMF. However a significant difference in weight loss experience was seen between genders, which prove to be in line with the study of Kuvat *et al.* reported nutritional imbalance plus weight and fat losses should be expected in female patients having double-jaw osteotomies, which are followed up with inter-occlusal splints after surgery (13).

In conclusion, intermaxillary fixation in orthognathic treatment does not result in a difference self-reported loss of body weight compared to no IMF. However, approximately one-third off all male patients and two-thirds of all female patients report weight loss after osteotomies. Further investigation is needed to assess the actual post-operative weight loss and changes in nutritional status in OGS patients and the relationship between changes in body weight, nutritional status and post-operative complications. These data are of utmost importance in developing proper treatment protocols for these patients. Treatment protocols should include pre- and post-operative dietician consultations and possible indications for medical nutrition and vitamins.
